# The Epidemiological and Spatiotemporal Characteristics of the 2019 Novel Coronavirus Disease (COVID-19) in Libya

**DOI:** 10.3389/fpubh.2021.628211

**Published:** 2021-06-14

**Authors:** Mohamed A. Daw, Abdallah H. El-Bouzedi, Mohamed O. Ahmed

**Affiliations:** ^1^Department of Medical Microbiology & Immunology, Faculty of Medicine, University of Tripoli, Tripoli, Libya; ^2^Faculty of Biotechnology, University of Tripoli, Tripoli, Libya; ^3^Department of Microbiology and Parasitology, Faculty of Veterinary Medicine, University of Tripoli, Tripoli, Libya

**Keywords:** Libya, COVID-19, epidemiology, spatiotemporal analysis, dynamics, geography

## Abstract

COVID-19 is a global pandemic that has affected all aspects of life. Understanding its geographical and epidemiological characteristics has become particularly important in controlling the spread of the pandemic. Such studies are lacking in North African countries, particularly in Libya, which has the second largest area of any country in Africa and the longest coast facing Europe. The objectives of this study are to determine the epidemiological parameters and spatiotemporal patterns of COVID-19 and outline strategies for containing the spread and consequences of the pandemic. This comprehensive study included all the confirmed cases of COVID-19 since its emergence in Libya on March 24, 2020 until July 31, 2020. The epidemiological characteristics of COVID-19 were analyzed and the spatial dynamic trends were explored. Regional counts of weekly reported cases were used to characterize the spatial dynamics of COVID-19. A total of 3,695 confirmed cases of COVID-19 were recorded: 2,515 men (68.1%) and 1,180 women (31.9%), with a male-to-female ratio of 2.1:1. Ages ranged between 2 and 78 years. Older patients infected with COVID-19 were at a risk of higher disease severity and mortality. Broad geographic variability and spatiotemporal spread variation of the COVID-19 pandemic in Libya was observed, indicating a significant increase of COVID-19 spread starting in the middle of July 2020, particularly in the western and southern regions, although it was consistently reported in the central and eastern regions as well. Assessing the spatiotemporal dynamics of COVID-19 in the early stages of the epidemic is particularly important in understanding the pandemic spread. Such assessments are essential for designing effective prevention and control programs aimed at reducing the impact of the COVID- 19 pandemic, particularly in countries with limited resources.

## Background

The ongoing coronavirus disease (COVID-19) pandemic has reached each country in the world and no one can be considered safe. The pandemic affects all aspects of life, socially, economically, politically, and even morally. Since its emergence, countries and health authorities have responded comprehensively but differently (1). However, the impact may vary between and within countries, partly because of the degree to which control strategies are adopted and executed. Countries such as Sweden and Germany responded early and successfully. Others, such as Italy, Spain, and France, acted differently and thus it resulted in a high number of deaths (2–4). The impact was even worse in developing countries such as Iran and Brazil, as the early action was inappropriate and was influenced by local understanding (5, 6). Hence, the epidemiology and impact of COVID-19 varies greatly from one country to another. Understanding these epidemiological parameters has become particularly important for each country.

In Africa, the incidence of COVID-19 has varied considerably between countries, possibly reflecting variations in the volume of air travel and differences in SARS-CoV-2 testing (7). Tackling COVID-19 has become increasingly difficult in northern and sub-Saharan countries, where the effects of internal armed conflicts and the emergence of other viral epidemics on the economy and health structures are still being felt (8, 9). Only a few African states have been successful in implementing detection, prevention, and control measures. Yet the COVID-19 pandemic poses a challenge not only for fragile African countries but also for those with well-functioning health systems. Until now, studies evaluating the epidemiological and spatial spread of COVID-19 in Africa have been limited. Understanding the spread of the pandemic is critical for predicting local outbreaks and developing public health policies during the early stages of COVID-19 (10, 11).

Libya, the second largest country in Africa and with the longest coast on the Mediterranean, facing Europe, has been involved in an armed conflict since 2011. The country is considered vulnerable to the spread of infectious diseases, including COVID-19. Armed conflicts and internal instability challenge disease control and have a very deleterious effect on the provision of health services (12, 13). Due to the low levels of international commerce and travel in the country, the seeding of COVID-19 came later than in other North African countries. The first few cases of COVID-19 identified in Libya arrived in March 2020 (14). Now that COVID-19 has taken a strong hold in the country, displaced people and immigrants can help spread it from one city to another (15, 16). Accordingly, COVID-19 is likely spreading rapidly in Libya but is to a large extent undetected by the health authorities. Understanding the epidemiological manifestations and local variation in the dynamics of the pandemic is a crucial step for developing more effective strategies for mitigating the risk of infection in vulnerable communities. Unfortunately, to date, there is no global standard response to the pandemic and each country is facing the crisis based on its own possibilities, expertise, and hypotheses (17).

Different studies have analyzed the epidemiological manifestations and geographic mapping of COVID-19 (10, 18). Such information is particularly important not only for controlling COVID-19 but also for planning to ameliorate the consequences of the epidemic. However, there is a lack of information on the epidemiology and clinical features of COVID-19 patients in North Africa and particularly in Libya. The objectives of this study were to evaluate the epidemiological and spatiotemporal distribution of COVID-19 in Libya and to highlight strategies for appropriate allocation of the healthcare resources to combat the spread of the pandemic.

## Materials and Methods

### Patient Information and Data Collection

The National Center for Disease Control in Libya performs laboratory tests for SARS-CoV-2, investigations, contact tracing, and quarantine at the regional or district level. We collected information provided by the Center on the demographics, epidemiological information, clinical symptoms, and outcomes from all laboratory-confirmed cases of COVID-19 initially suspected/identified by symptoms or through contact tracing all over the country between March 24, 2020 and July 31, 2020. The data of all the registered patients were collected, extracted from the hospital records, and checked by a clinical epidemiologist. Furthermore, we collected the countrywide, daily-updated number of laboratory tests for SARS-CoV-2 and their results, which were done at a rate of about 2,000 samples per day.

### Case Definitions

The definitions of the confirmed cases of COVID-19 were based on our previous publication (14, 19–21): a patient with evident clinical symptoms of COVID-19 and with a positive Nucleic Acid Amplification Test. The clinical severity of the disease was categorized as follows: (1) mild (only mild symptoms without evidence of pneumonia and not requiring oxygen therapy); (2) moderate (fever, respiratory tract symptoms, and imaging evidence of pneumonia); (3) severe (respiratory distress and respiratory rate of 30 per min in the resting state, finger oxygen saturation of 93%, and arterial blood oxygen partial pressure [PaO_2_/oxygen concentration (FiO_2_) of 300 mmHg (1 mmHg = 0.133 kPa)]. Critical cases were defined as those exhibiting respiratory failure and requiring mechanical ventilation, with the occurrence of septic shock, and admission to an intensive care unit with multiple organ dysfunction/failures. The pandemic spread was traced weekly (epi-weeks), which is a standard method for comparison of data during epidemic spread.

### Statistical and Geographic Analysis

The epidemiological characteristics of confirmed cases of COVID-19 were analyzed descriptively using computer software (StataCorp. 2013 version 11.0. Stata Statistical Software Release 13. College Station, TX: StataCorp LP). Spatiotemporal analysis and geographic mapping of COVID-19 cases was carried out using GraphPad Software as previously described (22–24). Briefly, the geographic coordinates were recorded at the centers of the enumeration areas based on the geo-referenced information of the patients. The corresponding national standard geo-codes at the provincial, city, and county levels were included in the analysis to identify the location of the reported cases.

## Results

The study population consisted of all confirmed cases of COVID-19 reported in Libya by July 31, 2020 (12:00 a.m.) 2020. A total of 3,695 cases were reported, and their epidemiological and clinical characteristics are illustrated in Table 1. Of these cases, 2,515 were men (68.1%), with a male-to-female ratio of 2.1:1. Ages ranged from 2 to 78 years.

**Table 1:** Epidemiologic and demographic characteristics of 3,695 confirmed cases of COVID-19 infection in Libya.

		**Survived**	**Died**	**Total**	***P* value**
**Demographic characteristics**		**3,621**	**74**	**3,695**	
		***n*** **(%)**			
	Male	2,462 (68)	53 (71.6)	2,515 (68.1)	<0.001
	Female	1,159 (32)	21 (28.4)	1,180 (31.9)	0.01
**Age group**					
	≤ 15	86 (2.38)	0 (0)	86 (2.3)	0.01
	16–20	142 (4)	0 (0)	142 (3.8)	0.01
	21–25	161 (4.5)	1 (1.4)	162 (4.5)	0.02
	26–30	241 (7.5)	0 (0)	241 (6.5)	0.01
	31–35	271 (7.5)	0 (0)	271 (7.3)	0.01
	36–40	307 (9)	3 (4.1)	310 (8.4)	0.01
	41–45	327 (9)	5 (6.8)	332 (9)	0.01
	46–50	350 (9.5)	7 (9.5)	367 (10)	0.01
	51–55	379 (10.5)	9 (12.2)	388 (10.5)	0.01
	56–60	398 (11)	13 (17.6)	409 (11.1)	<0.001
	61–65	427 (11.8)	15 (20.3)	442 (12)	<0.001
	≥ 66	541 (14.9)	21 (24.4)	562 (12.2)	<0.001
**Source of infection**					
	Imported	751 (20.2)	31 (42)	782 (21.2)	0.01
	Local	2,870 (79.3)	43 (58)	2,913 (78.8)	<0.001
**Clinical severity**					
	Mild	2,327 (64.3)	3 (4.1)	2,330 (36.1)	0.01
	Moderate	1,101 (30.4)	7 (9.5)	1,108 (30)	0.01
	Severe	102 (2.8)	26 (35.1)	128 (3.5)	<0.001
	Critical	91 (2.5)	38 (51.4)	129 (3.5)	<0.001
**Geographic region**					
	Western region	1,732 (47.8)	23 (31.1)	1,755 (47.5)	<0.001
	Middle region	418 (11.5)	11 (14.9)	429 (11.1)	0.01
	Southern region	1,102 (30.4)	31 (41.9)	1,133 (30.7)	<0.001
	Eastern region	729 (20.1)	9 (12.2)	738 (20)	0.01

Only 74 patients (2%) died. The case fatality rate was higher among men (53; 2.1%) than women (21, 1.2%). Of the deceased patients, 39 (52.7%) were aged ≥ 55 years (particularly >66 years). Only 5 patients (6.6%) were under 40 years of age, and 21 patients (28.4%) were 41–55 years old. Of all the cases, 782 (21.2%) were imported, and 2,913 (78.8%) cases were acquired locally (*p* ≤ 0.001). The imported cases were mainly from Egypt (257, 32.9%), Turkey (219, 28%), Tunisia (209, 26.7%), and Saudi Arabia (96, 12.3%).

The western region contributed the largest fraction of infections (1,755, 47.5%), followed by the southern region (1,133, 30.7%), the eastern region (738, 20%), and the central region (429, 11.1%). However, mortality rates were highest in the southern and central regions (2.7 and 2.6%, respectively) and lowest in the western and eastern regions (1.3 and 1.2%, respectively).

Of all the confirmed cases, 2,368 (64.0%) were mild, 1,108 (30%) were moderate, 128 (3.5%) were severe, and 91 (2.5%) were critical. The highest mortality rates were observed among the critical and severe cases: 38 (51.4%) and 26 (35.1%), respectively (*p* < 0.001). It was much lower among the moderate (7, 9.5%) and mild cases (3, 4.1%).

**Figure 1:**
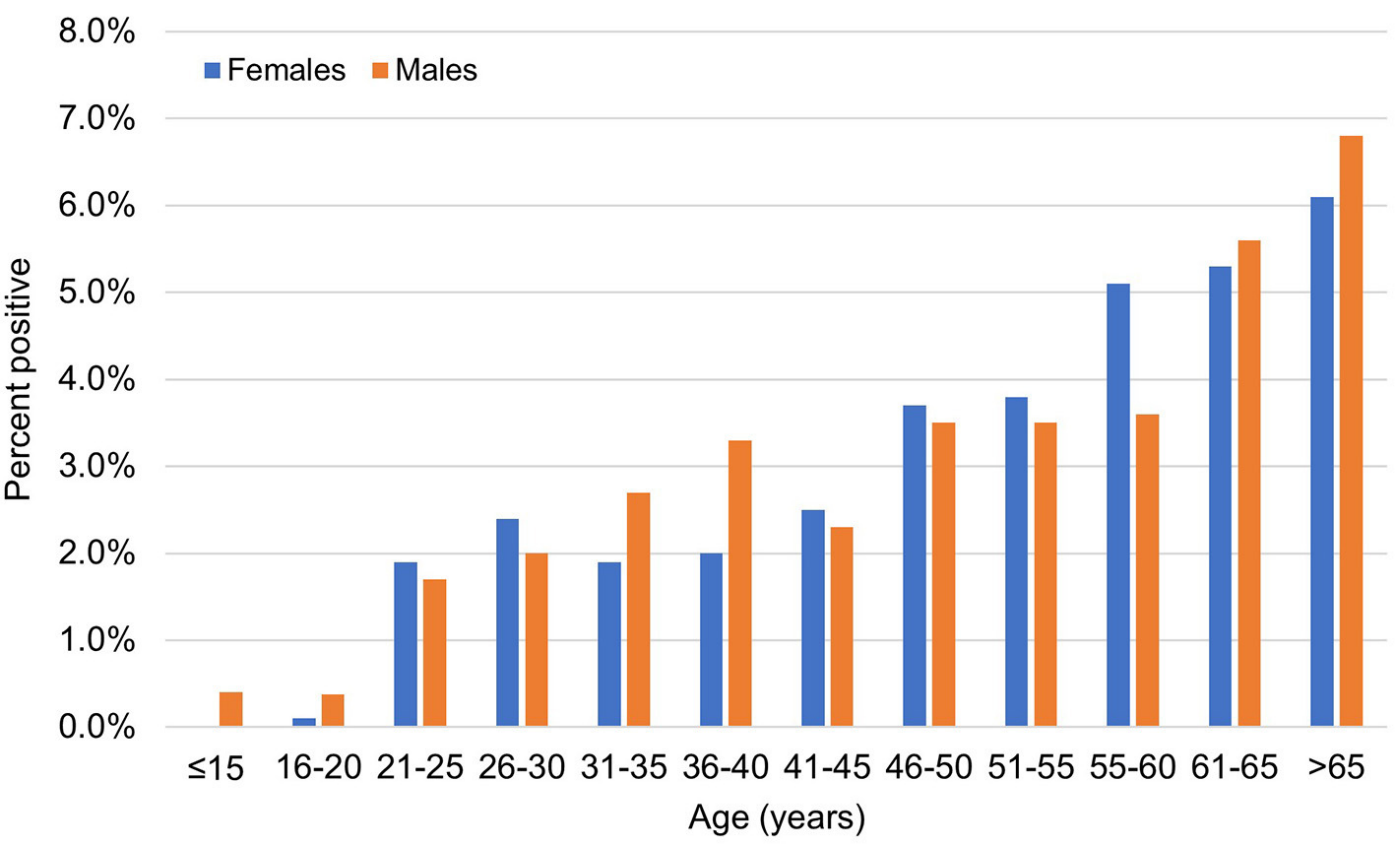
The age and sex distribution of confirmed cases of COVID-19 infections during the study period. Men (blue bars) and women (red bars).

**Figure 2:**
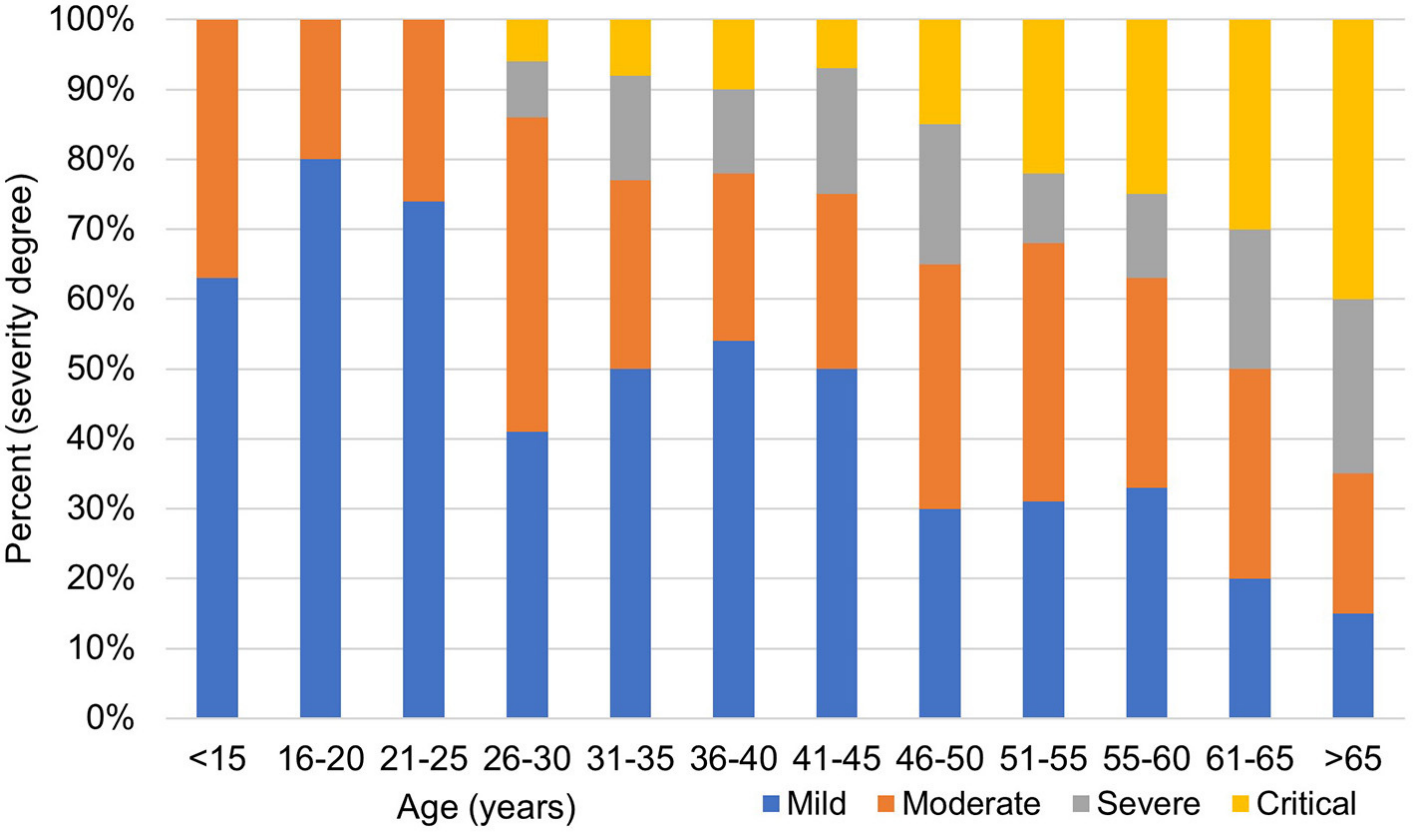
The clinical severity of confirmed cases of COVID-19 infection.

The age distribution of men and women is shown in Figure 1. The median age of the infected individuals was 55 years; 26.7% were aged ≥ 60 years and only 2.4% were <15 years. The occurrence of infection increased progressively with age, with men showing higher rates except for the oldest age group (>65 years) (*p* ≤ 0.001). This difference was also significant among patients above 50 years of age. The number of infected cases was higher among male patients (68%), indicating that COVID-19 tended to be more serious in men according to the clinical classification of severity. The association between illness severity and age is shown in Figure 2: illness severity increased with age. Most mild cases were among those aged below 45 years, followed by moderate cases. Most severe and critical cases were among older patients, particularly those aged ≥ 60 years (*p* < 0.001), who accounted for 45 deaths of mild cases (44.1%) and 51 severe cases (56%).

**Figure 3:**
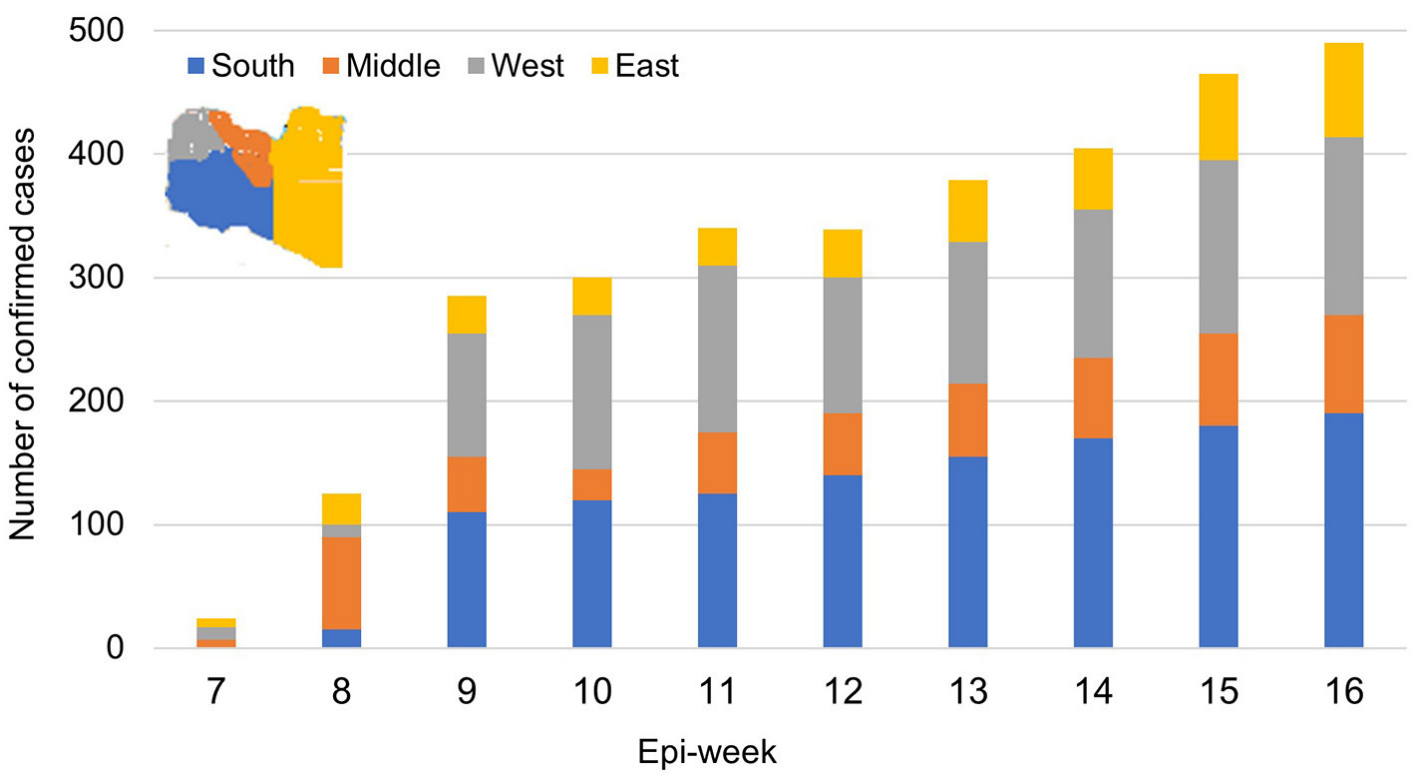
The weekly incidence trends of confirmed cases of COVID-19 in each Libyan region during the study period.

**Figure 4:**
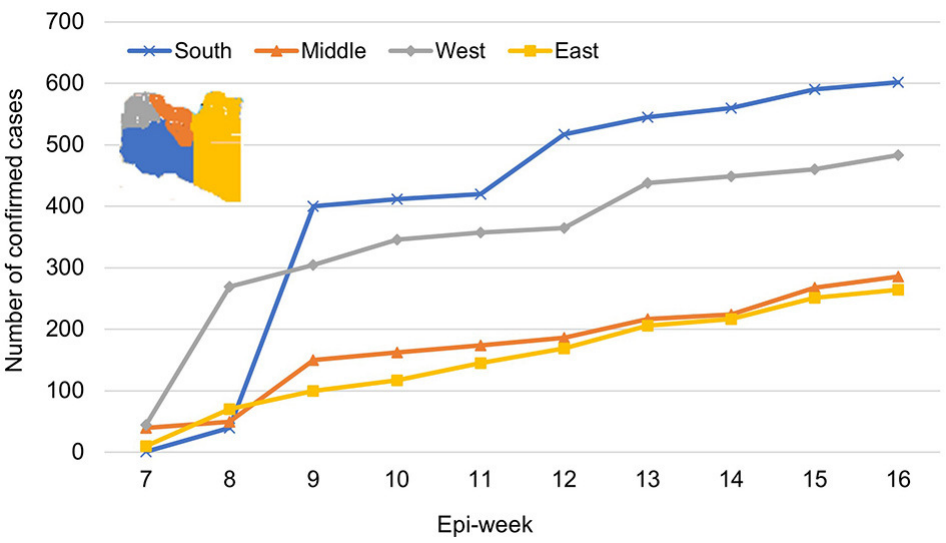
The prevalence of COVID-19 infections among the Libyan population in the four geographic regions over 16 epi-weeks (April 03–July 31, 2020).

Figure 3 shows the overall temporal trend of weekly counts of newly confirmed COVID-19 cases in the four Libyan regions. Infections were sporadic until early May (first 9 epi-weeks); 120 confirmed cases were reported, mainly in the western region (97). The number of weekly confirmed COVID-19 cases subexponentially increased across the country from the 10th to the 17th epi-week, followed by a slow decrease. During the entire observation period, the highest number of cases was reported in the western region (47.8%), followed by the southern region (30.4%), middle region (30.4%), and eastern region (20.1%) (Figure 4). However, during epi-weeks 9–16, the proportion of confirmed cases decreased in the eastern and central regions, but increased substantially in the southern region (Figure 4).

**Figure 5:**
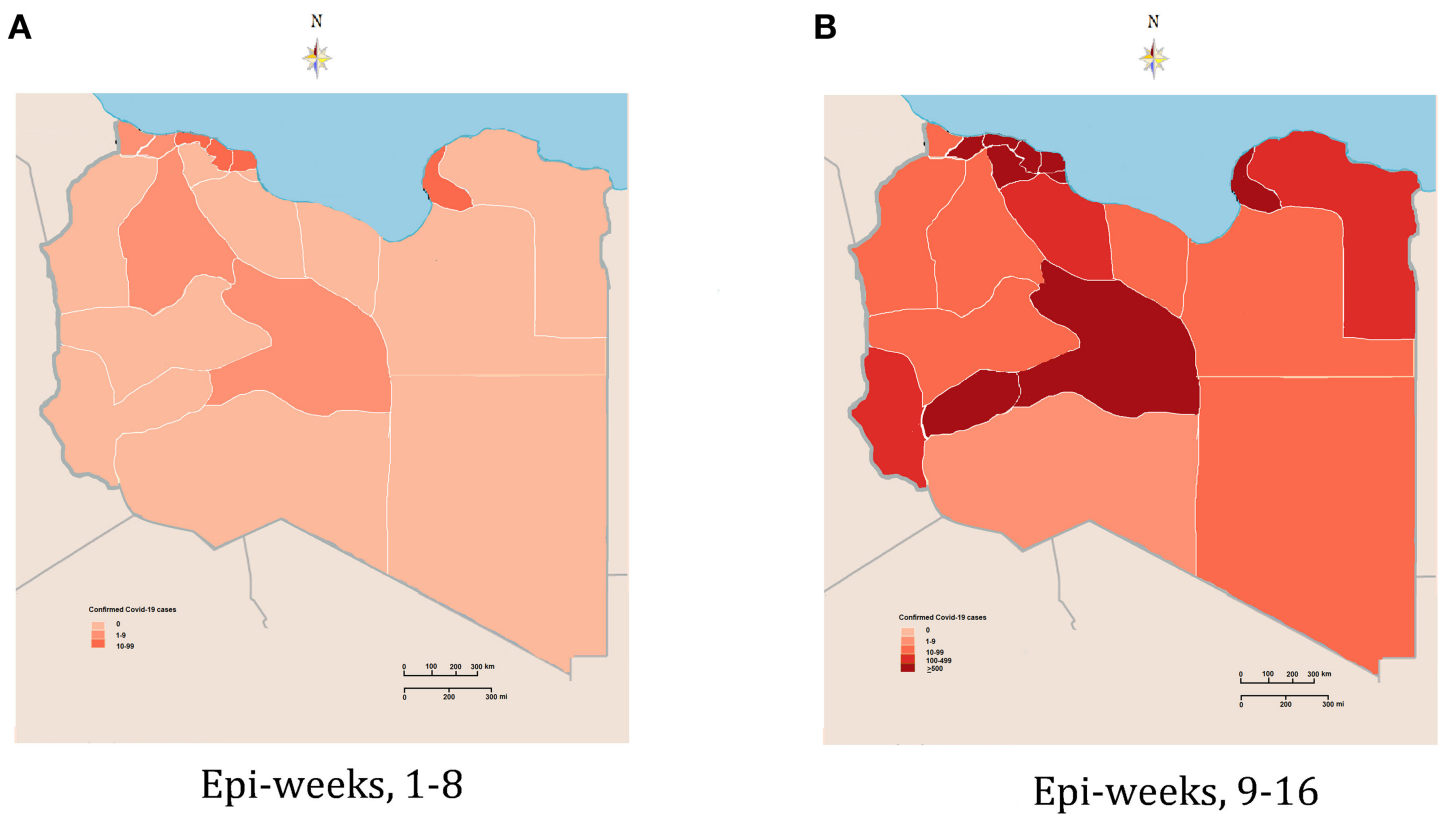
Geographic and spatiotemporal distribution of the confirmed cases of COVID-19 in Libya. **(A)** Early stage of the epidemic (epi-weeks 1–8). **(B)** Second stage of the epidemic (epi-weeks 9–16).

Spatiotemporal analysis and geographic mapping showed a marked geographic and temporal variation of COVID-19 cases during the pandemic period, as shown in Figure 5. In the first eight epi-weeks, the emergence of the pandemic was detected in five counties in the western region, including Tripoli, which hosted the largest number of cases, followed by Zawia, Surman, Aljalaet, and Nalut. Clustering analysis showed that new clusters emerged in the 9th epi-week, largely in the southern and western regions (*p* ≤ 0.001). In the following epi-weeks, the epidemic spread all over the country and new cases were reported in each of the 22 counties (Figure 5B). The cases were spatially distributed with agglomeration characteristics (Figure 5). The increase in the number of infections in one city will inevitably lead to increases in adjacent cities, which means that a positive spillover effect occurs.

## Discussion

The epidemiological and clinical features of the COVID19 pandemic in Libya are characterized in this study. By July 31, 2020, 3,695 cases were reported, representing 6.2/10,000 of the population, and the overall death rate of infected individuals was 1.3%. Median age was 55 years and the male to female ratio was 2.1:1. Our data show that COVID-19 infected men more than women; these findings are in concordance with other studies reported from China and Iran (25, 26). This indicates that sex and gender disparities are involved, or even sociocultural factors, particularly in the Middle East and Africa, and points to the need to gain a better understanding of the impact of sex and gender on the incidence and case fatality of the disease and to tailor treatment accordingly. However, other studies have shown that susceptibility to SARS-CoV-2 does not differ between men and women (27). In Libya, geographic differences were obvious. In particular, the southern region, which has the smallest population, contributed 30.7% of all infections.

Taking patient age into consideration, children (<15 years) accounted for only 2.4% and those aged 20–50 years accounted for 9.0% of all infections. Those aged over 60 years represented the largest fraction of infected cases, accounting for 20%. Clearly, COVID-19 among Libyans corresponds with higher age. In Italy, Spain, and France, most deaths in infected individuals occurred in elderly people suffering from severe conditions, particularly in the early phases of the epidemic (28, 29).

Based on our data, most of the reported cases were mild (2,368, 64.0%) or moderate (1,108, 30.0%). Only 128 patients (3.5%) had severe illness, and 91 (2.5%) were critical. The association between illness severity and age was evident. This study has shown that men tend to have more serious illness than women. This is in agreement with other studies carried out by Jin et al. (19) and Li et al. (30). Therefore, male sex may be considered as a risk factor for higher severity and mortality in patients with COVID-19, independently of age.

In this study, we evaluated the spatial and temporal patterns of the COVID-19 pandemic in Libya during the first 16 epi-weeks of the epidemic. In the early stage of the COVID-19 outbreak, a few sporadic cases were reported in first six epi-weeks in Tripoli in the western region. By the end of the 8th epi-week, the infections had spread to cities neighboring Tripoli, such as Musrata and Zawia. Since then, the number of weekly confirmed COVID-19 cases exponentially increased across the country from the 9th to the 16th epi-week. During the entire study period, the prevalence of COVID-19 in the Libyan regions showed striking geographic differences, with many more infections in the western and southern regions. This likely depended on the arrival of the first wave and the population movements in the regions (31, 32).

It is clear in this study that the dynamics of the epidemic in Libya followed a geographical differentiation, with a strong western to southern gradient. This indicates that an increase in the number of infections in one city may lead to increases in adjacent cities. Further, studies are needed to shed light on this speculation. However, similar trends have been observed in the early stages of the spread of COVID-19 in Italy, Spain, and France. Hence, specific strategies should be implemented to contain the expansion of the pandemic (33, 34).

Though the study gives detailed information on the epidemiology of COVID-19 in Libya, there might be some uncertainties about how well the reported data represent reality because many asymptomatic or mild cases go undetected. Another limitation is that the study may not highlight the impact of the armed conflict, which has stopped only recently, on the spread of COVID-19 in Libya and the ability to trace and identify infections in some cities and towns (35–38).

## Conclusion

This study is the first to provide information on the epidemiological characterization and spatial and temporal patterns of the COVID-19 outbreak during the first wave of the pandemic, which started in Libya in March 2020. The epidemic has involved the whole country, with infection rates varying from one region to another. Meanwhile, the prevention and control of COVID-19 in Libya still face an uphill struggle. The study demonstrated the spatiotemporal characteristics and trends of COVID-19 in Libya, which is essential for focusing preventive efforts. Hence, swift action to control further spread of the virus and to improve the response capabilities is urgently needed.

## Data Availability Statement

The data presented in this paper are freely available upon request.

## Ethics Statement

The Medical Ethics Committee at the Faculty of Medicine, University of Tripoli approved the study and waived the need for approval by the Libyan National Ethics Committee. As the study was an analysis of epidemiological data obtained at a national population level, it needed no consent from the participants.

## Author Contributions

MD conceived and designed the study, wrote the paper, designed the analysis, analyzed the data, and performed cartography. MD and AE-B contributed to the analysis tools. AE-B and MA made substantial contributions to conception and design, acquisition of data, or analysis and interpretation of data. MD, MA, and AE-B provided advice and critically reviewed the manuscript. All authors read and approved the final manuscript.

## Conflict of Interest

The authors declare that the research was conducted in the absence of any commercial or financial relationships that could be construed as a potential conflict of interest.

## References

[1] Haffajee RL Mello MM. Thinking globally, acting locally—The US response to COVID-19. New Engl J Med. (2020) 382:e75. 10.1056/NEJMp2006740 32240580

[2] Gianicolo E Riccetti N Blettner M Karch A. Epidemiological measures in the context of the COVID-19 pandemic. Deut Ärztebl Int. (2020) 117:336. 10.3238/arztebl.2020.0336 32527379

[3] Spiteri G Fielding J Diercke M Campese C Enouf V Gaymard A. First cases of coronavirus disease 2019 (COVID-19) in the WHO European Region, 24 January to 21 February 2020. Eurosurveillance. (2020) 25:2000178. 10.2807/1560-7917.ES.2020.25.9.2000178 32156327 PMC7068164

[4] Harbert R Cunningham SW Tessler M. Spatial modeling could not differentiate early SARS-CoV-2 cases from the distribution of humans on the basis of climate in the United States. PeerJ. (2020) 8:e10140. 10.7717/peerj.10140 33173618 PMC7594635

[5] Pourghasemi HR Pouyan S Heidari B Farajzadeh Z Shamsi SR Babaei S. Spatial modelling, risk mapping, change detection, and outbreak trend analysis of coronavirus (COVID-19) in Iran (days between 19 February to 14 June 2020). Int J Infect Dis. (*2*020) 98:90–108. 10.1016/j.ijid.2020.06.058 32574693 PMC7305907

[6] de Souza WM Buss LF Candido DDS Carrera JP Li S Zarebski AE. Epidemiological and clinical characteristics of the COVID-19 epidemic in Brazil. Nat Hum Behav. (2020) 4:856–65. 10.1038/s41562-020-0928-4 32737472

[7] Gilbert M Pullano G Pinotti F Valdano E Poletto C Boëlle PY. Preparedness and vulnerability of African countries against importations of COVID-19: a modelling study. Lancet. (2020) 395:871–7. 10.1016/S0140-6736(20)30411-6 32087820 PMC7159277

[8] Da'ar OB Haji M Jradi H. Coronavirus Disease 2019 (COVID-19): Potential implications for weak health systems and conflict zones in the Middle East and North Africa region. Int J Health Plann Manag. (2020). 19:10.1002/hpm.2982. 10.1002/hpm.2982 32557820 PMC7323081

[9] Gyasi RM. Fighting COVID-19: Fear and internal conflict among older adults in Ghana. J Gerontol Soc Work. (2020) 63:688–90. 10.1080/01634372.2020.1766630 32558630

[10] Daw MA El-Bouzedi AH Ahmed MO Cheikh Y. Spatial distribution and geographic mapping of COVID-19 in Northern African countries; a preliminary study. J Clin Immunol Immunother. (2020) 6:032. 10.1017/S0950268820001983

[11] Ngwira A Kumwenda F Munthali ECS Nkolokosa D. Spatial temporal distribution of COVID-19 risk during the early phase of the pandemic in Malawi. PeerJ. (2021) 9:e11003. 10.7717/peerj.11003 33665042 PMC7912604

[12] Daw MA. Libyan healthcare system during the armed conflict: challenges and restoration. Afr J Emer Med. (2017) 7:47. 10.1016/j.afjem.2017.04.010 30456107 PMC6234156

[13] Daw MA El-Bouzedi A Dau AA. The assessment of efficiency and coordination within the Libyan health care system during the armed conflict-2011. Clin Epidemiol Glob Health. (2016) 4:120–7. 10.1016/j.cegh.2015.07.004

[14] Daw MA. Preliminary epidemiological analysis of suspected cases of corona virus infection in Libya. Travel Med Infect Dis. (2020) 35:101634. 10.1016/j.tmaid.2020.101634 32205266 PMC7138189

[15] Daw MA. Corona virus infection in Syria, Libya and Yemen; an alarming devastating threat. Trav Med Infect Dis. (2020) 137:01652. 10.1016/j.tmaid.2020.101652 32247929 PMC7129830

[16] Daw MA El-Bouzedi AH Ahmed MO In Association with libyan study group of COVID-19. COVID-19 and African immigrants in North Africa: a hidden pandemic in a vulnerable setting. Disaster Med Public Health Prep. (2020) 19:1–2. 10.1017/dmp.2020 PMC7711340 33070803

[17] Rajendran DK Rajagopal V Alagumanian S Kumar TS Prabhakaran SS Kasilingam D. Systematic literature review on novel corona virus SARS-CoV-2: a threat to human era. Virus Dis. (2020) 11:1–3. 10.1007/s13337-020-00604-z 32656310 PMC7288266

[18] Daw MA El-Bouzedi AH. Modelling the epidemic spread of COVID-19 virus infection in Northern African countries. Travel Med Infect Dis. (2020) 35:101671. 10.1016/j.tmaid.2020.101671 32304743 PMC7159847

[19] Jin JM Bai P He W Wu F Liu XF Han DM. Gender differences in patients with COVID-19: Focus on severity and mortality. Front Public Health. (2020) 8:152. 10.3389/fpubh.2020.00152 32411652 PMC7201103

[20] Organization WH. Laboratory Testing for Coronavirus Disease 2019 (COVID-19) in Suspected Human Cases: Interim Guidance. Geneva: World Health Organization. (2019). Available online at: https://apps.who.int/iris/bitstream/handle/10665/331329/WHO-COVID-19-laboratory-2020.4-eng.pdf (accessed March 2, 2020).

[21] Organization WH. Clinical Management of Severe Acute Respiratory Infection When Novel Coronavirus (nCoV) Infection is Suspected: Interim Guidance, 25 January 2020. Geneva: World Health Organization (2020).

[22] Daw MA Daw AM Sifennasr NE Draha AM Daw AA Daw AA. Spatiotemporal analysis and epidemiological characterization of the human immunodeficiency virus (HIV) in Libya within a twenty five year period: 1993–2017. AIDS Res Therapy. (2019) 16:1–9. 10.1186/s12981-019-0228-0 31238947 PMC6591977

[23] Daw MA Daw AM Sifennasr NE Draha A Daw A Daw A. The epidemiological characterization and geographic distribution of Hepatitis D virus infection in Libya. Pan Afr Med J. (2020) 35:120. 10.11604/pamj.2020.35.120.20055 32637018 PMC7320781

[24] Daw MA Ali LA Daw AM Sifennasr NE Dau AA Agnan MM. The geographic variation and spatiotemporal distribution of hepatitis C virus infection in Libya: 2007–2016. BMC Infect Dis. (2018) 18:594. 10.1186/s12879-018-3471-4 30466399 PMC6251168

[25] Zhang J Yang S Xu Y Qin X Liu J Guo J. Epidemiological and clinical characteristics of imported cases of COVID-19: a multicenter study. BMC Infect Dis. (2021) 21:406. 10.1186/s12879-021-06096-6 33941096 PMC8090926

[26] Shahriarirad R Khodamoradi Z Erfani A Hosseinpour H Ranjbar K Emami Y. Epidemiological and clinical features of 2019 novel coronavirus diseases (COVID-19) in the South of Iran. BMC Infect Dis. (2020) 20:1–2. 10.1186/s12879-020-05128-x 32552751 PMC7301075

[27] Klein SL Dhakal S Ursin RL Deshpande S Sandberg K Mauvais-Jarvis F. Biological sex impacts COVID-19 outcomes. PLoS Pathogens. (2020) 16:e1008570. 10.1371/journal.ppat.1008570 32569293 PMC7307725

[28] Ceylan Z. Estimation of COVID-19 prevalence in Italy, Spain, and France. Sci Total Environ. (2020) 729:138817. 10.1016/j.scitotenv.2020.138817 32360907 PMC7175852

[29] Daw MA El-Bouzedi AH. Trends and projection of demographic indices of the Libyan population using a fifty-year census data 1954-2016. Afr Popul Stud. (2019) 33:4876–90 10.11564/33-2-1401

[30] Li Q Guan X Wu P Wang X Zhou L Tong Y. Early Transmission dynamics in Wuhan, China, of novel coronavirus-infected pneumonia. N Engl J Med. (2020) 382:1199–207. 10.1056/NEJMoa2001316 31995857 PMC7121484

[31] Sun F Matthews SA Yang TC Hu MH. A spatial analysis of COVID-19 period prevalence in US counties through June 28, 2020: Where geography matters? Ann Epidemiol. (2020) 52:54–9.e1. 10.1016/j.annepidem.2020.07.014 32736059 PMC7386391

[32] Rose-Redwood R Kitchin R Apostolopoulou E Rickards L Blackman T Crampton J. Geographies of the COVID-19 pandemic. Dialog Hum Geogr. (2020) 10:97–106. 10.1177/2043820620936050

[33] La Maestra S Abbondandolo A De Flora S. Epidemiological trends of COVID-19 epidemic in Italy over March 2020: From 1000 to 100 000 cases. J Med Virol. (2020) 92:1956–61. 10.1002/jmv.25908 32314804 PMC7264625

[34] Mavragani A. Tracking COVID-19 in Europe: an infodemiology study. JMIR Public Health Surveill. (2020) 6:e18941. 10.2196/18941 32250957 PMC7173241

[35] Daw MA El-Bouzedi AH Ahmed MO Alejenef AA. The epidemiological characteristics of COVID-19 in Libya during the ongoing-armed conflict. Pan Afr Med J. (2020) 37:219. 10.11604/pamj.2020.37.219.24993 33520058 PMC7821789

[36] Daw MA El-Bouzedi AH Ahmed MO. How are countries prepared to combat the COVID-19 pandemic during the armed conflict? the case of Libya. Travel Med Infect Dis. (2021) 40:101977. 10.1016/j.tmaid.2021.101977 33486055 PMC7826110

[37] Irwin A. How COVID spurred Africa to plot a vaccines revolution. Nature. (2021). 10.1038/d41586-021-01048-1 [Epub Ahead of Print]. 33883714

[38] Daw MA Daw AM Miftah MM El-Bouzedi A Ahmed MO Libyan Study Group of COVID-19 (LSG-COVID-19). Familial clustering and reinfection with 2019 novel Coronavirus (COVID-19, SARS-CoV-2) in the Libyan Community. Disaster Med Public Health Prep. (2021) 1–3. 10.1017/dmp.2021.68 [Epub Ahead of Print]. 33678217 PMC8134895

